# The influence of the Fourth Industrial Revolution on organisational culture: An empirical investigation

**DOI:** 10.3389/fpsyg.2022.919157

**Published:** 2022-11-24

**Authors:** Shwetha Singaram, Claude-Hélène Mayer

**Affiliations:** Department of Industrial Psychology and People Management, College of Business and Economics, University of Johannesburg, Johannesburg, South Africa

**Keywords:** higher education, organisational culture, COVID-19, Fourth Industrial Revolution, South Africa

## Abstract

The Fourth Industrial Revolution (4IR) is known to transform and create opportunities for the world of work. However, little is known about how the future workforce, such as university students, are being equipped and exposed to 4IR technologies and ways of thinking in a South African (SA) context. This study’s findings contribute to understanding the influence of organisational culture on the uptake of 4IR technology within higher education (HE) in SA during a pandemic. The study uses Edgar Schein’s theoretical framework to explore the organisational culture at a university in the Gauteng province. The article responds further to the questions on how 4IR technology and principles are understood and applied within the context, and how to investigate to what extent the 4IR is reflected upon or embedded in the university’s culture. A qualitative research design is used, and data are gathered through in-depth, semi-structured interviews from seven purposively selected academic and senior management staff members. Thematic analysis uncovered that the university’s ambitious and competitive culture contributed to a positive uptake of 4IR technology and principles, even pre-COVID-19. Furthermore, the specific influence of the university’s Vice-Chancellor to build 4IR thinking into the university helped shape more 4IR thinking and technologies, such as artificial intelligence, whilst still considering the existing disparities of SA, as a developing country.

## Introduction

Klaus Schwab, considered to be the father of the 4IR, referred to the 4IR as an expansion of the third industrial revolution and a unique revolution that the world has not experienced before (Schwab, 2017). Schwab (2017) suggested developing an all-inclusive and universal understanding of how technology influences our lives and alters our economic and socio-cultural environments. However, since Schwab’s announcement of the 4IR, Western or developed contexts’ perspectives have dominated the literature on understanding and shaping how 4IR is applied (Mayer and Oosthuizen, 2021; Mayer et al., 2021a,b). The developed world’s experience and access to technology are more advanced than in the developing world as developing countries are dealing more extensively with various challenges that are connected to poverty and inequality (Mayer and Oosthuizen, 2021; Mayer et al., 2021a,b).

The use of 4IR technologies have come a long way since the onset of COVID-19, especially in SA’s higher education sector (Mhlanga and Moloi, 2020). The exponential rise in positive COVID-19 cases globally led to the rapid shift to online learning. This urged universities to transform their teaching and learning methodologies to enable students to study and achieve their learning outcomes from home. In SA, some universities were able to adapt better than others (Mhlanga and Moloi, 2020). This could be attributed to the extent of digital literacy amongst staff and students, as digital literacy is cited as a basic need for students to adapt and participate in the new digitised society (Rusdah and Sutarsih, 2021). Considering the difficulties faced by graduates to be adequately skilled for a 4IR workplace, Kamaruzaman et al. (2019) highlights that the HE sector is influential in empowering graduates with appropriate skills and mindsets for the 4IR world. In this dynamic context, there is a need for higher educational institutions (HEI) to assist emerging graduates and remain globally competitive, as much as corporate organisations (Mwangi and Waithaka, 2018). Awareness and understanding of the organisational culture in a HEI can help achieve organisational goals, analyse the organisation, compare organisations, and unify all organisation members (Mwangi and Waithaka, 2018). Research indicates that a well-managed and appropriately developed organisational culture positively impacts organisational performance and the individuals within the organisation (Warrick, 2017; Mwangi and Waithaka, 2018).

A lack of a theoretical framework on the 4IR and the exponential rate of change means that it is difficult to anticipate how technological advancements will genuinely impact the world of work and the success of organisations and individuals (Newman, 2019). This is a reality for developing countries with limited resources and specific social challenges, such as the socio-economic inequality in SA. There has been limited research in the SA context on how HEI’s respond to the demands of the 4IR compared to international universities. The SA context requires a more localised perspective to respond to the 4IR that considers the socio-economic-cultural context of the country. This research intends to look at both the negatives and positives, challenges and opportunities, of the 4IR in HEIs in SA to contribute to a holistic perspective on what the 4IR may mean to the HE sector in this country.

This study focuses on the higher education context, looking at the organisational culture in a higher education institution (HEI). HEI’s are considered one of the most critical elements of global competition due to their ability to influence and change social development, science, technology, and economics (Köse and Korkmaz, 2019). Organisational culture is an effective way to understand how universities perform and are managed and is one of the critical factors to distinguish one university from another (Mwangi and Waithaka, 2018; Köse and Korkmaz, 2019). According to the Department of Higher Education and Training in SA, SA’s HEIs are responsible for developing and empowering students with the necessary skills for social and economic development (Wiseman et al., 2016). The success of these institutions is dependent on the performance of both students and academic staff, influenced by the institution’s culture (Wiseman et al., 2016). This study intends to examine and understand the influence of the 4IR on the organisational culture at a university in Gauteng, SA, from the perspectives of academics and senior management, providing education and leadership. To do this, the research responds to questions on how the organisational culture at the university is experienced and understood by the sample organisation members, how the 4IR is defined, understood, and applied at this university, and what influence the 4IR (technology and principles) has on the organisational culture at the university. This could serve as a contribution to greater research that needs to be done into how to effectively adapt to the 4IR and implement more innovative techniques within HEIs within developing countries, such as SA.

## Organisational culture and the 4IR

Organisational culture has proven to be an effective instrument to improve performance and agility (Gaus et al., 2019), which makes it a significant determinant of how the principles of 4IR are affecting the organisation (if at all) and how it is applied across the university’s levels of functioning. Thus, this research aims to explore the perspectives of academics and senior management within a specific faculty in a SA HEI on the 4IR and its influence on the university’s organisational culture. Most of the economic and technological disruptions in the last century were founded in an industrial revolution (Hirschi, 2018). Each industrial revolution is characterised by some technological advancement that significantly impacts manufacturing or production (Ślusarczyk, 2018). It can be noted that the technological advancements within all the industrial revolutions thus far have originated within a first-world or in a developed context, in either the USA or Europe (Xu et al., 2018; Popkova et al., 2019; Nankervis et al., 2021). This presents potential challenges for appropriately applying these technologies in a developing context with unequal access to resources and inequitable wealth distribution.

Data in the context of the 4IR is considered a necessary, natural resource or raw material that enables the other 4IR technologies to function and prosper (Xing et al., 2018). A literature review on 4IR reveals the following technologies as characteristic of this revolution, artificial intelligence, Internet of Things (IoT), robotics, 3D printing, machine learning and cyber-physical systems (Schwab, 2017; Xing et al., 2018; Anshari, 2020). The total use and implementation of the various 4IR technologies will differ across different contexts (Anshari, 2020). 4IR technologies rapidly change how people communicate, consume, produce, employ, and learn worldwide (Tsekeris, 2019). The technologies offer opportunities or threats for economies, societies and, more specifically, the workforce (Schwab, 2017). While the 4IR technologies are recognised for their innovation and increasing efficiency and accuracy, there is an ongoing debate regarding the extent to which the technologies are intended to optimise the human workforce to perform better or replace the human factor completely (Anshari, 2020).

Kamaruzaman et al. (2019), believe that the degree of influence the 4IR will have on jobs depends on the skill level of workers, where the appropriateness of existing abilities to meet the new demands on the workforce brought by the technological developments of the 4IR will be critical. Xing et al. (2018) emphasise that the human workforce is not redundant in the 4IR era but requires additional training to survive and thrive. The future of work is dependent on adaptive workforces; thus, adaptive, and flexible minds will be a necessary skill for employability (Gleason, 2018). Economic growth is primarily driven by knowledge production, and HEI’s are considered vital role players of knowledge production *via* research and training a competent workforce (Jung, 2020). Thus, there are many motivating factors to understand the 4IR in an educational context to determine what can be done to manage and reduce the gap between knowledge and skills, or a significant portion of the emerging workforce risk becoming redundant.

The 4IR had a significant impact on higher education during COVID-19. Innovative technology has made a significantly positive impact in many industries and the economy, yet the education sector has been hesitant to transform teaching and learning practices with 4IR technology (Mhlanga and Moloi, 2020). The 4IR presents opportunities and challenges for the education sector, with a specific impact on curricula, teaching and learning (Kayembe and Nel, 2019; Mbandlwa, 2021). Even though the 4IR had begun its transition into society’s functioning since the beginning of the 21st century, the full use of technology for teaching and learning has been restricted to supporting traditional approaches, such as project material and shared learning on virtual platforms (Oke and Fernandes, 2020; Modise and Van den Berg, 2021).

The successful integration of 4IR into HE requires appropriate skills to engage with the different technologies (Kayembe and Nel, 2019). Digital literacy is cited as a basic need or pre-requisite for students to adapt and survive in the new digitised society (Rusdah and Sutarsih, 2021). Whilst there is extensive research on the use and application of 4IR and associated technologies in manufacturing sectors, there is limited research on the perspectives of 4IR staff and students as end-users in the African education sector (Oke and Fernandes, 2020; Mbandlwa, 2021). This gap has limited the application of 4IR technologies and the enhancement of stakeholders’ experiences, in the SA education sector.

A literature review of technology-enhanced teaching and learning in the SA HE sector over 20 years (1996–2016) was conducted by Ng'ambi et al. (2016). Studying all 22 HEIs in SA, Ng'ambi et al. (2016) categorised SA’s technology enabled HE journey into four phases and compared this with international progress during the same period. The findings from this review highlight the limitations in the progress of technology enabled HE among SA’s HEI’s and between SA and more developed countries. This is mainly due to unequal access to resources and education, caused by a legacy of poverty and political discrimination.

There is a need to adapt teaching and learning at HEI’s to better equip the emerging workforce to meet the new demands of the 4IR era and create them actively (Penprase, 2018; Modise and Van den Berg, 2021). To create a more innovative and agile workforce in a developing context such as SA, careful consideration of access to resources and levels of digital literacy amongst the SA university student population needs to be made, and significant provisions are necessary to bridge the digital divide in our country. This digital divide within HEI’s has been brought into greater scrutiny by the pandemic COVID-19.

## COVID-19 and organisational culture

The COVID-19 pandemic led to the rapid digitisation of the education system in SA, and most other countries, through the mediums of online lectures, e-textbooks, virtual classrooms, and communication platforms (Qazi et al., 2020). The United Nations Educational, Scientific and Cultural Organisation (UNESCO) reported that over 91% of the world’s student population were affected by these closures (Oyediran et al., 2020). The swift spread of the virus and move to online learning systems meant that HEIs had to work under extreme pressure to adapt generally and in terms of new methodologies, with not enough attention given to how the HEIs and the diverse stakeholders would react and cope (Kerres, 2020).

The COVID-19 pandemic revealed various vulnerabilities in educational systems worldwide, such as under-developed online teaching infrastructure, digitally under-skilled educators, the complexity of the home environment and the information gap (Ali, 2020). As much as the adoption of online learning systems was beneficial and considered to be a “lifeline for education during the pandemic” (Oyediran et al., 2020, p. 2), it played a discriminatory role in the education of disadvantaged students who could not afford the internet connection or appropriate infrastructure where needed (Qazi et al., 2020). Penprase (2018) argues that an effective 4IR educational strategy should consider its impact on the human condition, such as how people from various socio-economic backgrounds are affected, maintaining human rights, and developing a new inter-cultural understanding of the transformation that occurs in societies.

## Edgar Schein’s theory in the South African higher education context

Xing et al. (2018) argue that SA HEI’s will benefit from and drive the 4IR in the country. It is expected that HEIs in SA will encourage the implementation and integration of 4IR technology within the HEIs and use said technology to bridge the equality gap amongst students (Xing et al., 2018). This research will contribute to exploring the validity of this statement and how a SA university would successfully apply the innovative thinking associated with implementing 4IR technology in a way that does not discriminate amongst the student population. This study applied Edgar Schein’s theoretical framework on organisational culture. Culture is considered a mental and social process that is an essential determinant of people’s behaviour, mindsets, and activities in a particular context (Gaus et al., 2019). It has been extensively researched in various contexts and found to be a significant factor in an organisation’s success by acting as an effective tool for organisations to increase motivation, commitment, morale, mental health and productivity amongst its employees (Mayer, 2011; Warrick, 2017; Gaus et al., 2019; Köse and Korkmaz, 2019).

In a study done by Mwangi and Waithaka (2018) that explored organisational culture at a Kenyan university, the researchers state that Schein’s framework allowed for a richer understanding of cultural nuances and organisational issues that are not accounted for in many other models or theories. This study, therefore, adopted Edgar Schein’s theoretical framework for defining and exploring the organisational culture due to the deeper understanding to be gained. Edgar Schein states that culture can be analysed on three levels, ranging from what is explicitly seen and felt to more unconscious or embedded aspects (Schein and Schein, 2017). There are certain beliefs, values and norms that endure between these levels that are utilised by members to describe the culture to themselves and others (Schein and Schein, 2017). The first level of analysis refers to phenomena that can be seen, felt, and heard when examining a new group and their culture, known as artefacts. Artefacts can be analysed through observable behaviour, language, and documents that emotionally impact the observer (Schein and Schein, 2017).

The second level of analysis aims to uncover and understand the beliefs and values that guide an organisation and its members to behave and think the way that they do (Schein and Schein, 2017). This includes the organisation’s goals, strategies, and philosophies (Yilmaz, 2014). Whilst this level is still observable, gaining insight from group members is still recommended to understand the meaning of the beliefs or values (Hogan and Coote, 2014; Schein and Schein, 2017).

An essential component for a shared belief or value to become a shared underlying assumption is that the solution or actions derived from that belief/value need to consistently yield positive results for the organisation (Schein and Schein, 2017). This describes the third level of analysis. According to Schein and Schein (2017), these assumptions are based on the unconscious beliefs of group members and are reinforced as the organisation deals with external adaptions and internal integrations.

Edgar Schein’s framework stands out amongst other research on organisational culture because it considers differentiating and analysing organisational culture on multiple levels and not just as a single construct (Hogan and Coote, 2014). Schein’s model is seen to offer a practical approach to discover and evaluate components of an organisation’s culture (Yilmaz, 2014). This type of analysis is most appropriate to this study which aims to explore the organisational culture of the specific university in terms of the implementation and assimilation of 4IR technology and thinking on an individual level, from the perspectives of academics and university leadership within one faculty at the university. This cannot be achieved without studying the university’s organisational culture from multiple angles to develop a more holistic understanding.

An organisation’s culture has tremendous value and influence on the organisation and its members. Al Issa (2019, p. 45) refers to organisational culture as “the glue that holds the organisation together.” Culture can bring out the best or worst in an organisation and its members by either creating supportive and innovative work environments that people enjoy working in or a dysfunctional, unsupportive work environment where members feel constantly stressed and anxious (Warrick, 2017). This means that an organisation’s culture significantly influences how an organisation functions and determines culture. Simultaneously, the way an organisation functions has a significant impact on its culture. Thus, for an organisation to be successful, it needs both a robust and healthy culture and effective practices and leaders that build and sustain the culture (Warrick, 2017; Al Issa, 2019).

Organisational culture can serve as an organisation’s strategic asset as it enables many factors that lead to an organisation’s success, mainly due to its significant influence on members’ behaviours (Arokodare et al., 2019). It is directly influenced by the organisation’s context and the external environment (Cole et al., 2014). In addition to work behaviours and attitudes, organisational culture also affects the physical structure of organisations, such as the open-spaced office areas and access to knowledge and decision making within the organisation (Cole et al., 2014).

This study focuses on the higher education context, looking at the organisational culture in a higher education institution (HEI). HEI’s are considered one of the most critical elements of global competition due to their ability to influence and change social development, science, technology, and economics (Köse and Korkmaz, 2019). Organisational culture is an effective way to understand how universities perform and are managed and is one of the critical factors to distinguish one university from another (Mwangi and Waithaka, 2018; Köse and Korkmaz, 2019). According to the Department of Higher Education and Training in SA, SA’s HEIs are responsible for developing and empowering students with the necessary skills for social and economic development (Wiseman et al., 2016). The success of these institutions is dependent on the performance of both students and academic staff, influenced by the institution’s culture (Wiseman et al., 2016).

## Research methodology

Drawing on the theoretical framework of this research, Edgar Schein’s work on organisational culture, organisational culture is understood as a subjective experience and is unique to every organisation (Schein and Schein, 2017). To make sense of an organisation’s culture, one must engage with its members and find out how they make sense of it (Ekwutosi and Moses, 2013). Quantitative approaches to understanding organisational culture are limited, as they have fixed questions and themes that do not adequately create opportunities for open-ended answers and further probing of topics of interest (Cai, 2008). The qualitative research approach seeks a holistic, in-depth understanding of rich, unstructured, and contextual information (Ponelis, 2015). Qualitative methodology aids in understanding a complex reality that cannot be quantified and in finding meaning within a specific context from a holistic perspective (Queirós et al., 2017). Therefore, this research used a qualitative research approach to gain a richer understanding of how the 4IR is understood by academics and leaders at the specific university and how the principles of the 4IR are reflected in the organisation’s culture.

### Philosophical assumptions and phenomenological research strategy

This research applied the interpretivist paradigm, assuming that reality is constructed and should be uncovered from participants (Thanh and Thanh, 2015). Interpretivism considers various viewpoints to gain an in-depth understanding of multiple perspectives (Ponelis, 2015; Thanh and Thanh, 2015), whilst positivism seeks to make generalisable conclusions (Al Issa, 2019). In this study, multiple perspectives ranging from participants with strong influence and authority to participants with less authority were considered to arrive at a holistic understanding of the university’s organisational culture. It was recognised that the participants’ social context, being the university, had a significant influence in framing the university members’ views. Thus, social constructionism was applied as an additional paradigm in this study.

Phenomenological research is considered a valuable research tool in understanding people’s lived experiences concerning a phenomenon, as it seeks to describe, understand and decipher the meaning of a phenomenon by studying the perspectives and experiences of those who have engaged with it (Marques and McCall, 2005; Neubauer et al., 2019). In-depth interviews are commonly used in this research strategy to obtain detailed descriptions from participants (Marques and McCall, 2005). The phenomenological approach offers insight into ‘what’ was experienced by participants and ‘how’ it was experienced (Zolnikov and Furio, 2020). In this study, the researcher applied the phenomenological approach to understanding what the 4IR means to the participants in the selected university setting and how it is experienced through the university’s organisational culture specifically.

### Sample and sampling strategy

In this study, non-probability sampling was applied, and participants working as academics and management at the university in Gauteng were purposively approached. The study used the homogenous sub-type of purposive sampling methods whereby the participants are selected based on their possession of similar characteristics (Etikan et al., 2016). The study’s participants share the attributes of being employed by the same university in Gauteng and belonging to the same faculty.

This qualitative research focuses on a small sample of participants to gain more detail and increase the richness of the data (Farrugia, 2019). The sampling process was adjusted throughout the research process, and the size of the sample is usually dependent on when the data reaches saturation, meaning that the research question is adequately answered and no new information is being gained from participants (Fusch and Ness, 2015; Farrugia, 2019). Creswell (1998), as cited by Marques and McCall (2005), suggested that 3–10 in-depth interviews as the aim of phenomenological research is to describe the meaning of a phenomenon as experienced by a small group of individuals to ensure the richness of the descriptions. From this recommendation, the researcher aimed to interview between 5 and 10 participants and concluded with a sample size of seven as data saturation was reached.

The sample of seven participants consisted of two academics, one Head of Department, one Deputy Head of Department and three participants who made up the Deanery for the faculty at the selected sample university. The sample consisted of two female participants and five male participants. The inclusion criteria used for participants were that they had to be permanent staff members at the sample university in Gauteng for a minimum of 6 months to allow for sufficient exposure to the organisation’s culture.

### Data collection, data analysis and ethical consent

Semi-structured interviews were conducted with the sample to gather primary data. Semi-structured interviews encourage subjective responses from participants by following an interview schedule, allowing additional themes to emerge, and encouraging a flowing conversation to obtain rich experiences and meanings (Dearnley, 2005; Evans and Lewis, 2018). In this study, the interview schedule was scripted to answer the research questions and was based on Edgar Schein’s theoretical framework on organisational culture and the existing literature and trends on the 4IR with specific adaptation to the HE context in SA.

In-person or face-to-face interviews are the traditional way of collecting this type of qualitative data (Evans and Lewis, 2018; Archibald et al., 2019; Gray et al., 2020). However, due to the lockdown restrictions in SA due to the COVID-19 pandemic at the time, the researcher conducted the interviews virtually, using the Zoom platform. Whilst research into the full use of technology, specifically video conferencing on Zoom, to facilitate data collection in qualitative research is relatively new and limited; there is sufficient research to support the use of video conferencing platforms to gather rich qualitative data once the researcher and participant can a build good enough rapport (Archibald et al., 2019; Gray et al., 2020).

Once ethical clearance was gained for the study and permission was given from the relevant gatekeepers to conduct the research at the specific university, the researcher approached participants to voluntarily participate in the study *via* email. In the communication to potential participants to voluntarily participate in the research study, the researcher included details on what the study is about, why it was being conducted and how participants’ input will be used. Furthermore, participants were reassured about their personal, identifying information being kept confidential and thus that their responses would be kept anonymous. This contributed to ensuring that participants provided their voluntary, informed consent to participate in the study.

Once the seven participants gave their informed consent, semi-structured interviews were scheduled and conducted virtually with these participants from July to November 2020 using the Zoom platform according to the availability of the participant and the researcher. The interviews were audio-recorded to aid transcription, and any identifying information related to the participants were removed. The recordings were stored on a password-protected computer and only accessed by the researcher. The qualitative research data in this study was analysed using Braun and Clarke’s (2012) thematic analysis. Thematic analysis uses a logical process to identify, organise and provide insight into patterns of meaning within a data set (Braun and Clarke, 2012). Braun and Clarke (2006) encouraged that thematic analysis is considered a foundational method for qualitative data analysis since it involves core skills that can be applied to other qualitative research methods. In thematic analysis, the researcher serves as the instrument for analysis by perusing through the data and making decisions regarding coding, theming, and contextualising the data (Nowell et al., 2017). Braun and Clarke (2006) outline six steps or phases of thematic analysis that are carefully followed and applied in this study. These include, becoming familiar with the data through reading and re-reading through the transcripts, developing initial codes to define the data, grouping similar codes to form themes, refining the themes according to coded data pieces to ensure the meaning of the data is accurately captured, defining and naming the themes, and lastly, reporting on the themes (Braun and Clarke, 2012).

Thematic analysis was conducted on the verbatim transcripts from the semi-structured interviews conducted on the seven participants (hereafter referred to as P1-P7). From this analysis, three themes and 12 sub-themes emerged to describe the data set. The three themes include Organisational Culture, The Fourth Industrial Revolution (4IR), and The Impact of COVID-19, as presented in the Findings section.

Ethical considerations and ensuring that ethical protocols in a research study involving people as participants are followed is critical to protecting both participants and the researcher from harm and misconduct, which positively impacts the quality of the research study and trustworthiness of responses obtained (Swain, 2016; Feldman and Shaw, 2019). Three main ethical considerations were applied in the study, including informed consent, voluntary participation, and confidentiality. These are linked to the ethical principles of autonomy, beneficence and nonmaleficence (Stake, 2005). The criteria to ensure the trustworthiness of a qualitative research study include credibility, dependability, confirmability and transferability (Connelly, 2016). Steps were taken to ensure the quality of data and findings in this research study. The credibility of research refers to what extent the study’s findings can be considered valid (Connelly, 2016). The researchers ensured this by ensuring the participants’ responses were securely stored to avoid data corruption or tampering and by consulting with each other throughout the data analysis process to ensure interpretations of the data were true and free from individual bias. The dependability of the research study is achieved through the researchers’ transparency in documenting how the study was conducted, and how research findings were reported on (Noble and Smith, 2015). Thus, the researchers clearly expressed the procedure followed to conduct this study and report on its findings. Confirmability refers to whether the data and interpretations were rationally processed in the study so that they may be repeated (Cypress, 2017). The researchers met this criterion by recording each step of the data collection and analysis process with interview notes, transcripts and recordings and a codebook. And finally, transferability refers to the application of the study’s findings in other contexts or other researchers (Connelly, 2016). The researchers ensured the transferability of the research findings by providing in-depth descriptions of the research process, sample and context, and the study’s relation and relevance to existing literature within other settings.

## Findings

In the following, the findings regarding the three themes Organisational Culture, The 4IR, and The Impact of COVID-19 are presented. The themes included different sub-themes, as follows: 1. Organisational Culture: Artefacts, Espoused Values and Beliefs, and Underlying Assumptions. 2. 4IR: the emotional impact of the 4IR, the implementation/application at the university, university leadership’s drive and 3. COVID-19 in the SA context: online learning, impact on staff and university resources.

### Organisational culture

Aligned to Edgar Schein’s theoretical framework, the first theme, organisational culture, refers to the participants’ understanding of organisational culture and how it is represented and communicated within the university (Schein and Schein, 2017). Organisational culture appeared in the data 175 times and consists of the following sub-themes: Defining Organisational Culture, Artefacts, Espoused Values and Beliefs, and Underlying Assumptions. Participants describe organisational culture as the way an organisation conducts itself. P1, P3, P4, and P6 mentioned that it is the way an organisation “does things” or, according to P7, “gets things done.” P1 highlighted that an organisation’s culture includes “practices that may perhaps set them apart from other organizations.” Many participants also saw organisational culture as the relationships built within the organisation and people’s interactions. P2 stated, “organisational culture is based on relational links” and P5 highlighted, “it influences how people interact.” Most participants expanded on what influences organisational culture, namely, beliefs, values and strategic principles.

The artefacts, or observable aspects, of the University described by the participants included the university’s research output, national and international rankings, physical structure and facilities, communications to staff, feelings towards the Vice-Chancellor and university leadership, the university’s atmosphere and the caliber of staff contributing to the university’s outputs. Participants emphasised communication as a contributing factor to maintaining the organisation’s culture, as “continuous communication helps to emphasise what the university’s focus or values are” (P4) and “staff receive monthly emails from the Vice Chancellor’s desk that highlight the ongoings of the university, achievements and any current matters that he wishes to bring attention to” (P1). Participants 1, 3, 4, and 7 positively reflected on the research output or profile of the university, saying that the university’s “research output is something to be proud of” (P1) and that it is clear that the university “wants to be the best university, with high standard research being published in prestigious journals” (P3). However, the university’s emphasis on rankings does take some toll on staff. It creates a high-pressure environment and atmosphere at the university; as highlighted by P2, the university “places emphasis on university rankings, and there’s a pressure to move up the ladder.”

Participants emphasise the university’s focus on driving results and maintaining an ambitious and competitive culture that allows the university to reach international standards as the primary values that influence all organisation members. The participants highlighted that “to maintain the high ranking, the university needs to be competitive in everything they do” (P6) and that “this university has a very driven and ambitious culture that emphasizes innovation and making a difference” (P7). The participants’ perspectives on the university’s high rankings and excellent research output due to the ambitious and results-driven values within their culture represent the underlying assumptions of the university. Participants also acknowledged the influence of external and internal factors on the university’s culture to drive results and achieve its ambitious goals, especially over the past few years with the 4IR and more recently with the COVID-19 pandemic.

### The Fourth Industrial Revolution

This theme describes how the participants make meaning of the 4IR, how it makes them feel about their work, how they see it being implemented at the university, and the consequences this has for individuals, the university and the country. The 4IR appeared 161 times in the data and consists of the following sub-themes, defining the 4IR, the emotional impact of the 4IR, the implementation/application at the university, university leadership’s drive, and the SA context.

When defining the 4IR, all the participants referenced the influx of technology. Participants spoke about how “the 4IR is related to how technology is advancing” (P1) and it being “the acceleration of technology into the workplace” (P3). Participants mentioned some examples of these technologies and spoke of them as either an evolutionary process or disruptive. Participants spoke about the emotional impact of the 4IR on the participants’ professional lives and the university’s culture and expressed a variety of feelings towards the 4IR and how it is compromising on the ‘human factor’ of organisations. P1 said they find the 4IR “very scary” as it is taking over many human capabilities, whereas P3 stated that they have “always liked technology and not fearful of it” P2 remains “ambivalent” towards the 4IR, whereas P6 freely expresses that they are “very excited about the 4IR” and P4 feels good about the “positive application of technology to make work easier or better.”

Participants expressed how they see the 4IR technologies and principles being implemented or applied at the university. P7 highlighted that “a lot of the effort that we put into growing global excellence and stature has got a very strong tech-enablement, tech-empowerment dimension to it. So, there’s a huge amount of money being invested, and also a very strong strategic alignment. It’s become part of our culture.” P6 mentions that the university “has invested in state-of-the-art technology in their classrooms and in many places within the university you are able to access Wi-Fi for example.” In addition to infrastructure and culture, participants make reference to how the 4IR is being implemented into the university’s curricula, as the university is “trying to incorporate content into course modules that encourages innovative thinking in a changing environment” (P4) and the university aims to “produce courses or qualifications that are more contextualized and fit-for-purpose.” P5 elaborates that “the use of technology is aligned to teaching pedagogies or methodologies,” implying that the university is applying its discretion on what technologies to use and how. P4 supports this in saying that the university “uses own judgement and rationality to choose which technology is most suitable to their needs and not get overwhelmed with the multiple options.”

P3 shared how “‘Fees Must Fall’ prepared the university to take the 4IR route to prepare for similar future disruptions,” and this has become especially relevant during the COVID-19 pandemic. P7 supports this by mentioning that “the university has invested more in its computer-assisted learning endeavours since 2015” when the ‘Fees Must Fall’ campaign took place in SA. Despite the university’s seemingly swift adaption and implementation of 4IR technologies to provide for students, P7 acknowledges that “the university has been utilising 4IR technology well, but there is room for improvement.”

The implementation or application of the 4IR at this university is primarily influenced by the leadership’s drive to push the 4IR agenda. All the participants specifically referenced the vision of the current Vice-Chancellor (VC). P4 goes so far as to call the VC the champion of the 4IR at the university, “in my mind he is one of the champions for understanding the benefits of 4IR.” Although the momentum and passion for implementing the 4IR may have initially increased through the VC there has been significant uptake from staff and students since then. P6 mentions, “So, as you know the 4IR was a concept that was brought in by the current Vice-Chancellor but if you check you’ll see that lecturers are pushing in the right direction, in the same direction and students, like yourself, are doing studies on the Fourth Industrial Revolution.” Therefore, the concept and consequent behaviours related to the 4IR at this university were driven by the top-down but continued by all members within the organisation to become part of its culture.

During the phases of implementing the 4IR technologies and principles at the university, some participants highlighted how the level of inequality in our SA context has a significant role to play in the acceptance of the 4IR and the impact of its benefits; and the social responsibility of the university to contribute to closing that gap amongst its students. P1 highlights that universities must consider that “we are serving some of the poorest of the poorest in the country. So how do we navigate that space to make sure we are advancing?” P2 shared their concern that the 4IR was embraced too quickly, they are concerned that the increased application of 4IR technologies is “going to enhance inequality in our society rather than doing away with inequality” (P2). However, P6 was able to comment from a leadership perspective that the SA context is being considered in the stages of 4IR implementation at the university.

### The impact of COVID-19

The third theme encapsulates the impact of COVID-19 on the university and participants and how the university responded to the onslaught of the pandemic and lockdown restrictions on the HE sector in SA, using 4IR technologies and principles. This theme emerged 63 times in the data and consists of the sub-themes, online learning, impact on staff and university resources.

The participants recognised that the COVID-19 pandemic and full transition to online learning played a significant role in accelerating the university’s adaptation and application of technology to continue functioning at its prime. The university successfully and swiftly adapted to online teaching and learning with its already existing online platform. “The classroom facilities, technology used for teaching and the support within the university for learning, helped the university cope with the pandemic and shift online” (P5). P7 highlighted the increase in student performance, “I see this in the module success rates which have actually gone up by about 1.5% against all expectations, so we did not leave any student behind.” The university also had to adjust its policies to adequately deal with the various changes taking place.

However, these swift changes significantly impacted staff members who felt overwhelmed with the additional workload, new technology, and having to navigate their personal lives through the pandemic. P6 acknowledged from a leadership perspective that the many changes over the past year have “probably caused some psychological impact on the staff members. There has also been some emotional impact on the staff members.” The university did try to provide both staff and students with additional resources such as data to use the online learning platform. It offered training and support services to staff members on how to effectively engage with the online learning system in uploading material and administering assessments. “There was a lot of support that came from within the University. We have different support structures… they have developed a lot of resources for staff” (P5). P2 also highlighted that the staff were regularly sent surveys to check in on their wellbeing and how they were coping.

### Integrating the findings

Participants depicted the university’s culture as very results-driven, with the goal being global excellence. This perspective is based on what participants see, hear, and feel about the university and the influence of regular communications, leadership objectives, and strategy. The participants define the 4IR as an influx of new and advanced technologies that the university commonly uses to enhance administrative processes and accompany conventional teaching and learning. However, the arrival of the COVID-19 pandemic somewhat forced the university to optimise how they use 4IR technology to deliver online learning so that the academic year could continue successfully. This sudden shift to using semi-developed digital infrastructure allowed students to continue with the academic year but put large amounts of stress and pressure on staff members who had to adopt new teaching methods and adapt module content quickly. The impact on students’ experiences and the quality of learning were not considered in this study, but a rise in student performance was reported by participants. The digital inequality amongst SA university students was also considered, and whilst the university made arrangements to empower all students with mobile data, this provision of resources needs to be sustained in the university’s renewed policies and strategy going forward.

COVID-19 has brought both positive and negative consequences for this university, bringing opportunities to optimise learning experiences and ease of access and challenges to drive equality, ensure quality, sustain the appropriate resources, and support the wellbeing of staff.

## Discussion

In the following, the findings are reflected in light of Schein’s organisational framework.

### Organisational culture

Based on the findings, participants seem to understand organisational culture as how an organisation conducts itself and builds relationships between organisational members that is unique to that organisation and therefore differentiates it from others. This perspective concurs with how organisational culture is defined within Edgar Schein’s theoretical framework and the existing literature. In Edgar Schein’s framework, organisational culture is described as unique to every organisation and is based on shared meaning, behaviours, and learnings amongst organisation members (Schein and Schein, 2017). Martínez-Caro et al. (2020) refer to organisational culture as a means of competitive advantage and a key contributor to an organisation’s effectiveness. Furthermore, Al Saifi (2015) refers to organisational culture as a collection of values, behavioural norms, principles and practices shared by the individuals within an organisation.

Participants described what they saw, felt and heard about the university’s culture, also known as Artefacts (Schein and Schein, 2017), as its research output, rankings, physical structure and facilities. The perceptions about the university’s research output and rankings seem to be primarily informed by what is regularly communicated to staff members from the university’s leadership and what is observed in the university’s environment. This is consistent with other studies on organisational culture. Ramachandran et al. (2011) highlight that artefacts of an organisation’s culture can include buildings or office spaces and technology used. Organisational artefacts are identified as modes of communication about the organisation’s culture to internal and external stakeholders and can be leveraged to enhance the organisation’s identity both internally and externally (George et al., 2012). This directly relates to the participants’ favourable views on the university’s rankings and the positive recognition the university receives for its high-quality research output in various national and international journals.

Participants in this study highlighted the sometimes-negative impact on staff wellbeing caused by the pressures of such a results-driven culture at the university. The participants acknowledge the university’s competitive nature as a positive contribution to the university’s success but also hoped they would receive more support and resources, especially with regards to the changes over the past year due to the COVID-19 pandemic. A study on organisational culture during the COVID-19 pandemic reported that establishing psychological safety amongst organisation members is crucial in ensuring that cultural changes due to external events such as the pandemic are appropriately managed without resistance (Spicer, 2020).

### The Fourth Industrial Revolution

Participants in this study understand and describe the 4IR as an evolution of disruptive technologies that change the way things are traditionally done. The types of 4IR technologies referenced by participants include artificial intelligence, the Internet of Things and blockchain technology. This understanding of the 4IR’s impact correlates with the literature, which states that the 4IR technologies have swiftly changed processes, systems and the way people interact with each other and their environment (Tsekeris, 2019; Anshari, 2020; Mhlanga and Moloi, 2020). Furthermore, the references to 4IR technologies align with the literature, which refers to artificial intelligence, machine learning and the Internet of Things as characteristic technologies of the 4IR (Schwab, 2017; Xing et al., 2018; Anshari, 2020).

The participants expressed various positive and negative feelings towards the 4IR and its impact, such as fear and excitement. Some were fearful that a greater uptake and implementation of 4IR technologies would mean compromising on the human factor of organisations and the loss of human connection between staff and students at universities due to online learning. This opinion is shared by other researchers and studies that highlight the human consequences of automation being a loss of connection and, in some instances, unemployment of lower-skilled workers (Gleason, 2018; Kamaruzaman et al., 2019).

Findings from a study conducted by Baber (2020) considers the effect of social interactions on online learning, between staff and students, and amongst students in Korea found that social interactions increase the positive effects of online learning but are negatively affected by social distancing norms due to lockdown restrictions related to the COVID-19 pandemic. This argument is supported by a study done at a SA university that highlighted that despite the advantages of convenience and being able to watch a lecture repeatedly for better understanding, the lack of human interaction in online learning led to reduced levels of motivation and a lack of thorough understanding of complex concepts that require hands-on demonstration, amongst certain groups of students (Legg-Jack, 2021). It seems that a hybrid approach in HEIs may be more beneficial going forward, and universities would need to consult their staff, students, and resources to find an optimal balance between investing in their physical or digital infrastructure (Oginni et al., 2021).

When it came to implementing or applying the 4IR in this university, participants highlighted that a lot of effort and resources had been invested into growing the technology-related enablement and empowerment of university members, even before the pandemic struck. Other studies reference external forces that have driven the uptake and implementation of the 4IR in universities or organisations, with the majority referring to the COVID-19 pandemic as a “wake-up call” to invest in better technological platforms and resources (Baber, 2020; Mhlanga and Moloi, 2020, p. 4). However, this university acted on par with other SA and international universities who utilised technology and data to make a successful transition to online learning during the pandemic to avoid disruptions to the academic year (Mhlanga and Moloi, 2020; Legg-Jack, 2021).

Curricular and the components of teaching and learning are what make up the primary impact of the 4IR in the education sector (Schwab, 2017; Butler-Adam, 2018), and participants highlighted that the various university departments and faculties have been making an effort to integrate innovative thinking principles and future-fit strategies into their course content, to better equip students for the ever-changing and technologically empowered world beyond their university careers.

Online technologies form the foundation of the 4IR, and among the disadvantages of online learning are the challenges linked to access to the appropriate hardware and software to enable online teaching and learning, such as laptops or computers, data, and a stable internet connection (Legg-Jack, 2021). Many studies reference the disparities between universities in developed and developing countries and how that affected their response to the pandemic and transition online (Muftahu, 2020; Legg-Jack, 2021). These disparities were made more apparent by the pandemic, especially the digital divide in developing countries, such as SA (Makumbe, 2020; Oginni et al., 2021). Accordingly, participants acknowledged the unique challenges linked to the uptake of the 4IR and inequality in the SA context and emphasised the efforts being made to ensure that no student is left behind throughout the conceptualisation and implementation of the 4IR at the university.

### The impact of COVID-19

The COVID-19 pandemic significantly redefined the delivery of HE across the globe (Muftahu, 2020). Prior to the pandemic, most universities used online learning as a complement to traditional in-person learning; however, the pandemic has changed this to a new norm where online learning has become a vital role player in ensuring the continuation of teaching and learning in HEIs (Oginni et al., 2021). COVID-19 affected every university worldwide, in some way or the other, and despite having unequal access to resources, the pandemic offered HEIs in developing countries an opportunity to review their strategies and enhance student experiences with more significant investment in their facilities, staff and online resources (Green et al., 2020; Mogaji and Jain, 2020). This correlates with the participants’ reflections that the university efficiently transitioned onto their pre-existing online teaching and learning platform, which required some additional enhancements. The participants also expressed that the pandemic and online learning transition essentially forced the university and its staff to rapidly increase the utilisation and implementation of 4IR technologies into their teaching and learning practices. This opinion is shared in other studies, where the transition to virtual learning due to the pandemic is referred to as a “forced experimentation” that is encouraging universities around the world to enhance their technological infrastructure and resources (Sahu, 2020, p. 4).

The rapid transition and adaptations that had to be made were especially pressurising on staff and compromised their wellbeing within the ambitious and competitive culture at this university. This experience is shared by others, as the literature highlights that both staff and students within HEIs were vulnerable to psychological distress related to virtual or remote learning during the pandemic (Akour et al., 2020; Sahu, 2020; Chan et al., 2021). A study conducted at a Spanish university found high levels of depression and anxiety amongst its university members during the pandemic lockdown, which highlights that it is critical to monitor the wellbeing state of university members regularly and for universities to provide the appropriate psychological services to manage the emotional impact of the pandemic and online learning transition (Odriozola-González et al., 2020).

## Conclusion and recommendations

This study intended to explore and capture the influence of the 4IR on a university’s culture, using Edgar Schein’s framework for organisational culture, in terms of the uptake of 4IR technology and principles. Based on a review of the literature prior to collecting data for the study, the researcher expected that the implementation of 4IR technology and principles of innovation and efficiency would encourage the university to adjust its culture to be more innovative and agile. After collecting data for this study and analysing the responses from participants, it seems that this university’s culture was already innovative in driving results and seeking high international rankings. This, in turn, had a positive influence on the university’s inclination towards 4IR technology and principles and the application thereof.

Therefore, in this study, the university’s ambitious and competitive culture impacted the uptake of 4IR technology and principles at the university before the COVID-19 pandemic. Furthermore, the study’s findings indicate that the specific influence and motivation of the university’s Vice-Chancellor to convey 4IR into the university had already triggered policy-making and strategy renewal to incorporate more 4IR-aligned thinking and technologies, such as artificial intelligence, before COVID-19 and the national lockdown forced universities to transition to online learning and alter their teaching and learning practices, whilst still making considerations for the disparities within the SA context. These two findings emphasise the substantial influence of organisational culture and leadership in driving strategic outcomes and organisational behaviour, especially during a crisis such as the COVID-19 pandemic.

The researchers chose to focus on the perspectives of a purposively selected sample of academic staff members within a specific faculty at the university, which focuses the understanding of the university’s culture to only the selected sample’s views based on their exposure and experience. Therefore, additional studies may be conducted to gain the perspectives of staff and even students in other faculties across the university. The study used qualitative methodology to interview the sample of seven participants, thereby ring-fencing the rich data to a specific context and group of people. Combining the semi-structured interview strategy with a semi-structured or structured online survey may be helpful in reaching more participants, especially since data collection took place during a strict COVID-19 lockdown with restrictions on social interaction and movement.

This study focused on the perspectives of academic staff and management within the university, which limits the study to the exposure and experiences of that type of university staff member. Understanding a more diverse range of perspectives, such as from administrative staff and the university students would be beneficial for future studies. However, the study’s findings will meaningfully contribute to the discussion and body of knowledge regarding organisational culture, implementation of the 4IR and response to the COVID-19 pandemic with 4IR technology at a HEI in the SA context. This study’s findings have many practical implications. Firstly, the findings contribute to a limited body of literature on how universities in SA are applying the principles and technologies of the 4IR, aligned to the university’s organisational culture, before and during the COVID-19 pandemic. Secondly, the study offers insight into how academic staff members were affected by the rapid transition to online learning during the lockdown restrictions in SA related to the pandemic. The study also displays the critical influence of leadership, their vision and initiatives on organisational behaviour and culture, and implementation of new technology at a university. And finally, the study displays how an effectively sustained organisational culture can be leveraged, directly and indirectly, to drive new strategies, adapt to changes in the external environment and unify the vision and behaviour of organisational members.

## Data availability statement

The raw data supporting the conclusions of this article will be made available by the authors, without undue reservation.

## Ethics statement

The studies involving human participants were reviewed and approved by the University of Johannesburg, Department of Industrial Psychology and People Management Research Ethics Committee. The patients/participants provided their written informed consent to participate in this study.

## Author contributions

SS collected the data and wrote the manuscript. C-HM supervised the work. All authors contributed to the article and approved the submitted version.

## Funding

This work is based on the research supported by the National Research Foundation of South Africa (Grant Number: 123016).

## Conflict of interest

The authors declare that the research was conducted in the absence of any commercial or financial relationships that could be construed as a potential conflict of interest.

## Publisher’s note

All claims expressed in this article are solely those of the authors and do not necessarily represent those of their affiliated organizations, or those of the publisher, the editors and the reviewers. Any product that may be evaluated in this article, or claim that may be made by its manufacturer, is not guaranteed or endorsed by the publisher.
